# Transparent reporting on multi-site studies about algorithms for linked health data: A systematic review of Health Data Research Network Canada’s Algorithms Inventory

**DOI:** 10.23889/ijpds.v9i5.2800

**Published:** 2024-09-18

**Authors:** Lisa M Lix, Nasiba Ahmed, Daryl Fung, Saeed Al-Azazi, Naomi C Hamm

**Affiliations:** 1 University of Manitoba

## Objectives

Multi-site studies that use routinely-collected, linked health data often rely on validated algorithms to harmonize definitions of health conditions or other population characteristics across sites. If algorithm validation is not practicable, feasibility studies that assess whether an algorithm can be consistently and accurately implemented across sites, are recommended. We investigated the transparency of reporting on multi-site algorithm feasibility studies found in Health Data Research Network (HDRN) Canada’s Algorithms Inventory.

## Approach

Published, multi-site Canadian feasibility studies about chronic or infectious disease algorithms were assessed using an adaptation of the RECORD (REporting of studies Conducted using Observational Routinely collected health Data) and STROBE (STrengthening the Reporting of OBservational studies in Epidemiology) guidelines. Reviewers were trained on a subset of studies. The remainder were independently assessed by two reviewers and quality checks were conducted. Results were descriptively summarized.

## Results

Thirty published multi-site algorithm feasibility studies were reviewed; the majority were conducted in four or more sites, and 83% assessed chronic disease algorithms. Almost one-fifth (17%) of the studies did not justify the methods used to assess algorithm feasibility. However, the majority (>95%) addressed potential sources of bias and discussed algorithm generalizability.

## Conclusions

This study provided valuable insights into patterns of pedestrian and cyclist injuries among Peel residents, revealing considerable cross-regional healthcare utilization.

## Implications

Transparent reporting of algorithm feasibility studies facilitates algorithm reuse, enhances the credibility and reproducibility of study findings, and promotes collaboration within the data linkage community.

